# Histological improvement of liver fibrosis in well-treated patients with autoimmune hepatitis: A cohort study

**DOI:** 10.1097/MD.0000000000007708

**Published:** 2017-08-25

**Authors:** Åsa D. Borssén, Richard Palmqvist, Stergios Kechagias, Hanns-Ulrich Marschall, Annika Bergquist, Fredrik Rorsman, Ola Weiland, Hans Verbaan, Nils Nyhlin, Emma Nilsson, Mårten Werner

**Affiliations:** a Department of Public Health and Clinical Medicine; b Department of Medical Biosciences, Pathology, Umeå University, Umeå; c Department of Gastroenterology and Hepatology, Department of Medical and Health Sciences, Linköping University, Linköping; d Department of Molecular and Clinical Medicine, Institute of Medicine, Sahlgrenska Academy, University of Gothenburg, Gothenburg; e Department of Medicine, Section of Hepatology and Gastroenterology, Karolinska Institutet, Karolinska University Hospital Huddinge, Stockholm; f Department of Medical Sciences, Section of Gastroenterology and Hepatology, Uppsala University, Uppsala; g Division of Infectious Diseases, Department of Medicine, Karolinska Institutet, Karolinska University Hospital Huddinge, Stockholm; h Gastroenterology Division, Department of Clinical Sciences, Lund University, University Hospital Skane; i Department of Gastroenterology, Faculty of Medicine and Health, Örebro University, Örebro, Sweden.

**Keywords:** autoimmune hepatitis, autoimmune liver disease, cirrhosis, fibrosis, inflammation

## Abstract

Autoimmune hepatitis (AIH) is a chronic autoimmune liver disease that if left untreated may lead to the development of cirrhosis. Previous studies on AIH patients have suggested that fibrosis and even cirrhosis can be reversed by medical treatment. The aim of this study was to evaluate the efficacy of medical treatment for protection of developing fibrosis and cirrhosis.

A total of 258 liver biopsies from 101 patients (72 women, 29 men) were analyzed by a single pathologist and classified according to the Ishak grading (inflammation) and staging (fibrosis) system. Liver histology was stratified according to the temporal changes of fibrosis stage (increased, decreased, or stable), and groups were compared.

Complete or partial response to medical treatment was 94.9%. Reduction of fibrosis stage from the first to the last biopsy was seen in 63 patients (62.4%). We found an association between a reduction in the fibrosis stage and continuous glucocorticoid medication, as well as lowered scores of inflammation at last biopsy. Twenty-one patients had cirrhosis (Ishak stage 6) at least in one of the previous biopsies, but only 5 patients at the last biopsy.

Histological improvement is common in AIH patients that respond to medical treatment, and a reduction or stabilization of fibrosis stage occurs in about 2/3 of such patients.

## 1 Introduction

Autoimmune hepatitis (AIH) is a chronic autoimmune liver disease that if left untreated eventually may lead to the development of cirrhosis.^[1–3]^ Previous studies on AIH patients have shown that approximately 30% have cirrhosis already at diagnosis.^[4,5]^ Immune modulating treatment has improved the long-term outcome considerably for AIH patients.^[1,2,6]^ Successful treatment of AIH aims to minimize the hepatic inflammation, hence reducing the risk of progressive fibrosis and development of cirrhosis, consequently minimizing the need of a liver transplant and/or liver-related complications or death. Studies on other chronic liver diseases (hemochromatosis, nonalcoholic fatty liver disease, hepatitis B and C) have shown that fibrosis, and in some cases even cirrhosis, was reversed when the underlying condition was treated.^[7–10]^ Also, in AIH, previous studies have suggested that fibrosis can be reversed, but the point from which fibrosis is no longer reversible is still unknown.^[11–13]^

The objective of the present study was to study the course of inflammation and fibrosis in a large and well-defined cohort of AIH patients, and if fibrosis and cirrhosis are reversible when treating the inflammation. Since AIH is a fairly uncommon disease,^[1]^ the studied cohorts are usually small. In this multicenter study, we have collected one of the largest materials of repeated liver biopsies in AIH patients that has ever been studied.^[11–16]^

## 2 Methods

A well-defined cohort from all Swedish university hospitals was collected from 2001 to 2003.^[4]^ The cohort consisted of 473 patients (358 women and 115 men). Data from the in- and outpatient records were retrieved to identify as many AIH patients as possible, and approximately 1/3 of the Swedish AIH population was collected. An AIH score, originating from the international AIH group's criteria,^[17]^ but with modifications was calculated.^[4]^ Thus, the study originates from clinical data and all biopsies were obtained in a clinical setting. Data on laboratory values and medical treatment was collected at diagnosis, 6, 12, 24, 60, and 120 months, and at last follow-up.

**Figure 1:**
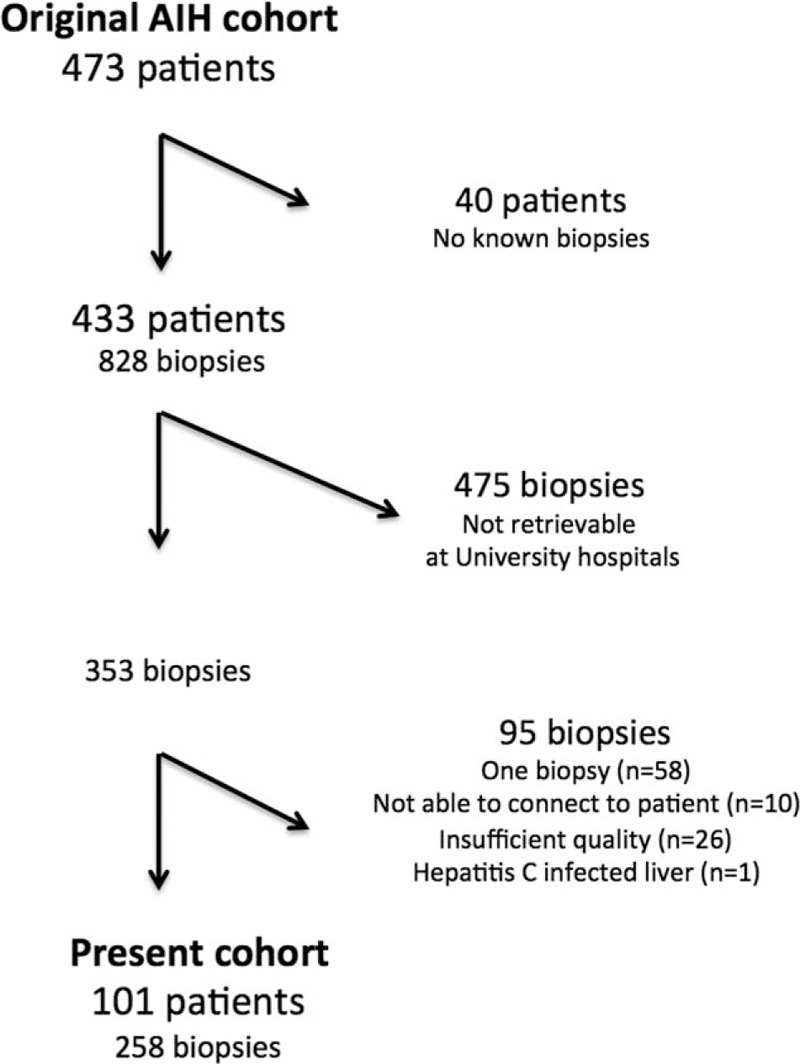
Flow chart of the inclusion of biopsies.

From the AIH cohort (n = 473), we identified 828 biopsies in 433 patients that were taken between 1963 and 2003. Only patients who had more than 1 biopsy were included to be able to follow the course of fibrosis. For practical reasons, only biopsies that were analyzed at large centers, that is, University Hospitals, were included. Eventually 353 biopsies were analyzed, but only 258 biopsies (from 101 patients) could be included in the study. The reasons for excluding 95 biopsies were most often that only 1 biopsy had been retrieved, but also that the quality of the biopsies was substandard, insufficient, or not possible to link to a specific patient (Fig. 1).

Histological evaluations were made by 1 single experienced pathologist (RP, co-author) in a conventional manner, but who was blinded for patient information such as treatment or outcome and the order of biopsies. Routinely stained sections according to each center's staining protocol were retrieved. Biopsy material encompassing at least 5 portal tracts or a length of at least 10 mm was considered suitable for interpretation. The biopsies were evaluated using light microscopy, and fibrosis stage and inflammatory activity were classified according to the Ishak score.^[18]^ A change of fibrosis stage from the first to the last biopsy was considered significant if the increase or decrease was 1 or more steps on the Ishak scale of fibrosis. For inflammatory activity, the change was considered significant if the change was 2 steps or more.

### 2.1 Statistical methods

A 2 sided *P*-value < .05 was considered statistically significant. Values are presented as median, per cent, and range where appropriate. For nonparametrical analyses, the Wilcoxon signed-rank test was used for comparing matched samples, Mann Whitney *U* for comparisons between 2 groups and Kruskal–Wallis for multiple groups. Categorical variables were evaluated by chi square-test and Fisher's exact test (for small samples). A logistic regression was performed investigating if sex, age, and inflammatory activity at diagnosis would affect the outcome. The statistical analyses were done in collaboration with a professional statistician, and SPSS version 21 (SPSS Inc., Chicago, IL) was used for statistical analyses.

### 2.2 Ethics

The committee for human ethics at Umeå University approved the project in 2005 (Dnr 04–174M), with an amendment in 2011 (2011/79 32M). Informed consent was not retrieved from the participants, but participants were informed orally by their physician and through an advertisement in the local newspaper. The risk of harm or identification was considered low. The study was conducted according to the 1975 Helsinki declaration of ethical guidelines.

## 3 Results

### 3.1 Demographics of the present cohort

A total of 258 biopsies (1979–2003) from 101 patients (72 women and 29 men) were analyzed. At diagnosis, women were in median 37 years of age (range 14–73 years) and men 24 years of age (range 12–73 years). The modified AIH score,^[4]^ calculated at the time of diagnosis, was in median 19 (range 10–25) with no statistical difference between men and women.

Sixty-four patients had undergone 2 biopsies (23 patients had 3 biopsies, 9 had 4 biopsies and 5 patients had 5 biopsies). Seventy-nine patients had biopsies taken at the time of diagnosis, and most biopsies were taken within 10 years after AIH diagnosis (Table 1). The median follow-up time between the first and the last biopsy was 3 years (range 0.5–23 years). An overlap between AIH and another autoimmune liver disease was suspected in 20 patients—primary biliary cholangitis (PBC) in 14 patients, primary sclerosing cholangitis (PSC) in 5 patients, and AIH-PBC-PSC in 1 patient.

**Table 1:**
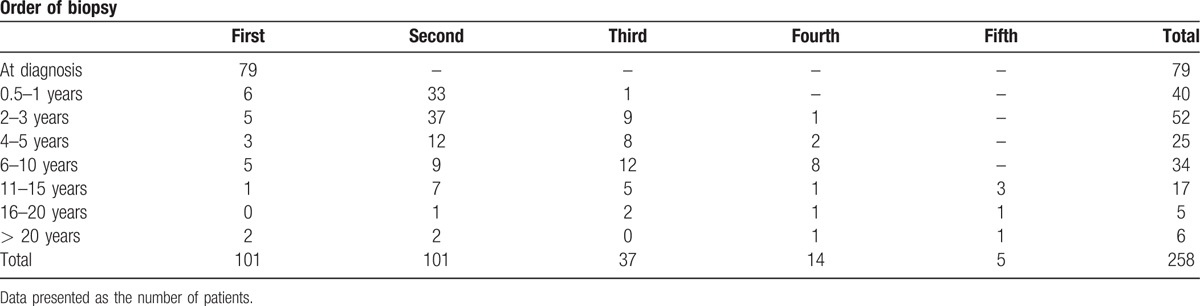
Order of biopsies (from first to fifth biopsy) and the year after AIH diagnosis the biopsy was taken.

Today, we have information on the status for 90 of the 101 patients. Eleven patients were partly lost for follow-up, but we know that they are alive and have not received a liver transplant in Sweden. Thirty-two of the remaining patients have died, of which 2 were liver transplanted. There are 2 patients alive after successful liver transplantations (in 2015). Thirty-nine patients are considered to be in remission without cirrhosis and 17 with cirrhosis, of which 2 are decompensated and one of them waiting for a transplantation.

### 3.2 Histological changes in inflammation and fibrosis

**Figure 2:**
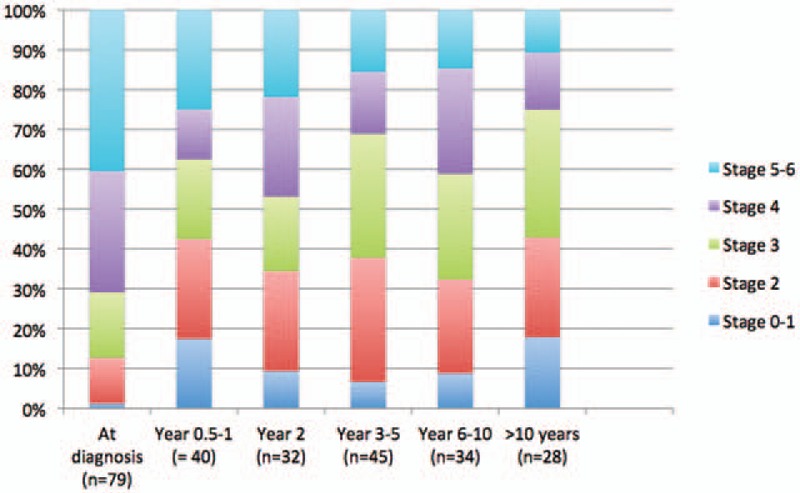
Fibrosis stage in 258 biopsies from 101 AIH patients, sorted by year after diagnosis. AIH = autoimmune hepatitis.

The most common outcome was a decrease of fibrosis stage, whereas an increase was the least common (Table 2). Evaluation of the index biopsy at AIH diagnosis always harbors a risk of overrating the fibrosis stage due to parenchymal collapse. Therefore, we also analyzed the fibrosis change excluding the index biopsies and biopsies taken within the first year after the diagnosis. We found the same trends, with the majority of patients showing reduction of fibrosis over time. Increased fibrosis was still the least common outcome if the criterion for a change in the fibrosis stage was expanded to 2 steps or more (Table 2). Figure 2 shows the stage of fibrosis related to when in time after AIH diagnosis the biopsy was taken.

**Table 2:**
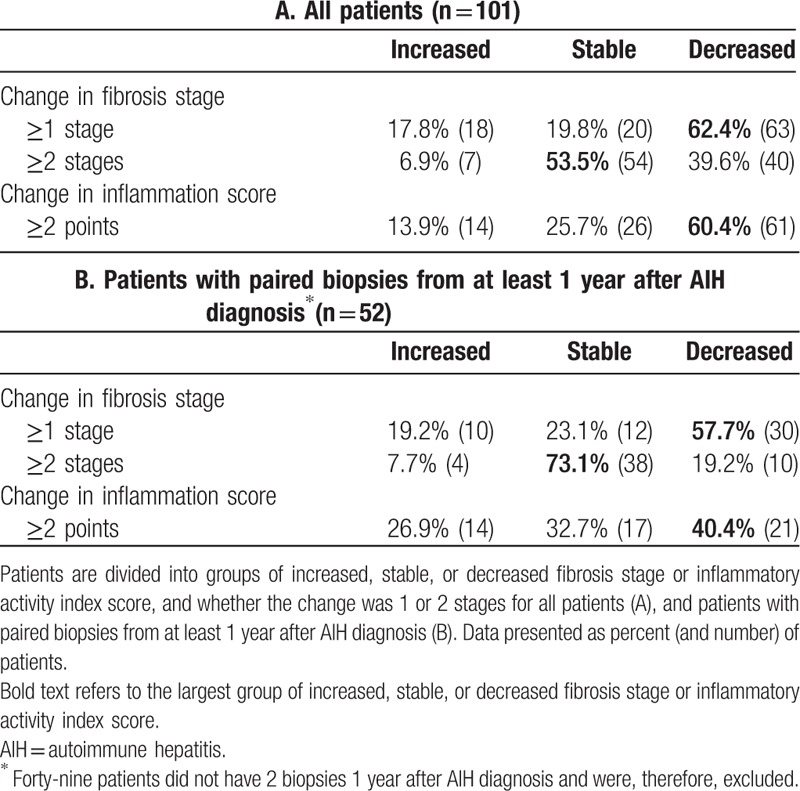
Change in the fibrosis stage and inflammation from the first to last biopsy.

The group with reduced inflammatory activity had the largest number of patients, both if starting at diagnosis or 1 year later (Table 2). Fifty-one patients achieved histological remission of inflammation at last biopsy, as defined by an activity index ≤3 at last biopsy. When evaluating only biopsies with an activity index ≤3 taken at least a year after AIH diagnosis, we found a significant difference between the groups with decreased, stable, and increased fibrosis stage (*P* < .001). The largest proportion of patients was in the group that had a decreased fibrosis stage (20 out of 27 patients, 74.7%). The median activity index score within the group with an activity index ≤ 3 was 2 (range 0–3) and median fibrosis stage was also 2 (range 0–6). When comparing groups with activity index ≤3 and >3 at last biopsy, the fibrosis stage was significantly higher in the latter (median 2 vs 3, *P* = .01).

### 3.3 Changes in inflammation and fibrosis and clinical outcome

There were no significant differences between patients that increased, decreased, or had a stable fibrosis stage in the frequency of liver transplantations, presence of cirrhosis or death until the end of 2015. There were also no differences in age at diagnosis, gender, and whether patients went into remission (complete or partial) or not. Further, there were no differences in the presence or absence of antinuclear antibodies (ANA), smooth-muscle cell antibodies (SMA), or antimitochondrial antibodies (AMA).

A logistic regression was performed investigating if sex, age, or inflammatory activity at diagnosis would affect a successful outcome—defined as a decreased or stable fibrosis stage and Ishak stage ≤4 at last biopsy. None of the analyzed parameters had an impact on the outcome.

### 3.4 Medical treatment

The frequency of patients receiving medical treatment in the cohort was high throughout the observational period. At some time, 98 patients (97%) had been on glucocorticoids and 82 patients (81.2%) had been on thiopurines. All patients but 2 who had medical treatment were on first-line medical therapies (glucocorticoids and/or thiopurines). Two patients had second-line therapy and were both treated conventionally before trying other medications.

In comparison between patients with increased and decreased fibrosis stage, there was no difference in treatment with glucocorticoids, but patients with a decreased fibrosis stage had fewer attempts to withdraw steroids (*P* = .002) (Table 3). This was not observed with thiopurines. It was also common to reintroduce both steroids and thiopurines after withdrawal.

**Table 3:**
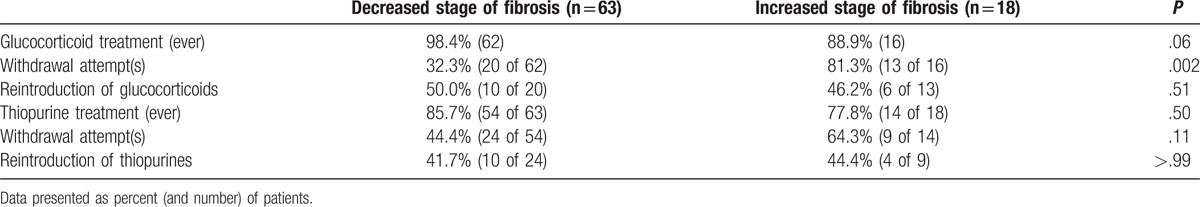
Patient groups with decreased and increased stage of fibrosis and the treatment with glucocorticoids and thiopurines, withdrawal attempts, and reintroductions.

Among all patients that had treatment with glucocorticoids, there was no statistical difference in stage of fibrosis at last biopsy between patients with and without withdrawal attempts; also, there were no differences in time between diagnosis and death. The coverage of data of long-term effects of treatment with glucocorticoids varied (45–83%). When comparing risk for long-term effects of treatment between patients with and without continuous glucocorticoid treatment (hypertension, development of Cushing, weight gain, cataract, diabetes, osteoporosis, and osteoporosis related fractures), there was no significant difference between the groups with or without withdrawal attempts.

### 3.5 Patients with biopsies at the time of diagnosis

Biopsies taken at diagnosis (n = 79) showed a higher fibrosis stage than at first follow-up biopsy (median 4 vs 3, *P* < .001). Furthermore, the inflammation score was higher at diagnosis than in the first follow-up biopsy (median 7 vs 4, *P* < .001). Fifty-eight patients (73.4%) had a decreased fibrosis stage from the first to the last biopsy. Eight patients (10.1%) had an increased and 13 patients (16.5%) a stable fibrosis stage, respectively. Patients who had a biopsy at the time of diagnosis were most often naïve for glucocorticoids and immune modulators at that time (62 patients, 78.5%). Among the treatment naïve patients, patients with a decreased fibrosis stage had higher alanine aminotransferase (ALT) (*P* = .039) at the time of the diagnosis compared to the groups with stable and increased stage of fibrosis. The group with an increased fibrosis stage had a significantly lower international normalized ratio (*P* = .017) compared to the other groups, but no significant difference was found for immunoglobulin G (Table 4).

**Table 4:**
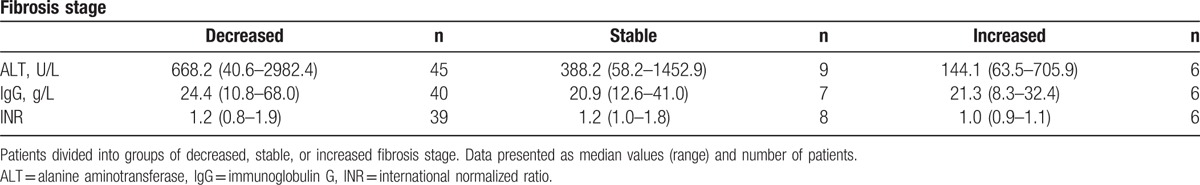
Laboratory values at the time of diagnosis for patients without treatment at the time of first biopsy, stratified according to outcome of change in the fibrosis stage at last biopsy.

Seven out of 79 patients with a biopsy from the time of diagnosis presented with an acute AIH with markedly elevated transaminases and Bilirubin. Six out of 7 improved the fibrosis from the first to the last biopsy and the last patient had a stable fibrosis over time. There were no statistical differences between patients with an acute and nonacute presentation over time regarding fibrosis stage or activity index score at last biopsy, or whether the fibrosis had increased, decreased, or remained stable.

The response to medical treatment was high; 94.9% had a complete or partial response (49 and 26 patients, respectively). Two patients did not respond to medical treatment, of which 1 eventually needed a liver transplantation. For 2 patients, the information on treatment response was missing. There were no differences in death or liver transplantations between patients with a complete or a partial response. Neither did we find any differences in fibrosis stage or activity index score at last biopsy, nor whether the fibrosis had increased, decreased, or remained stable.

### 3.6 Patients with cirrhosis

Twenty-one of the 101 patients had at least 1 biopsy with Ishak stage 6. In total, these patients underwent 57 biopsies (2–5), taken 0–20 years after AIH diagnosis. Seventeen of these patients had cirrhosis already in the first biopsy, with all but 1 taken at the time of diagnosis. There were no significant differences in gender distribution (male 23.8%, female 76.2%) compared to noncirrhotic patients. Most patients had a reduction in the fibrosis stage from the first to the last biopsy (15 patients, 71.4%). Even if biopsies taken within 1 year from diagnosis and the biopsies that lost their matched pair were excluded, a reduction of fibrosis stage was still the most often observed outcome (72.2%, n = 8). Thus, there were only 5 out of 21 patients who had stage 6 at their last biopsy. The inflammation score in the first collected biopsy was also higher in the cirrhotic compared to the noncirrhotic group of patients (median score 8 vs 6, *P* = .001).

## 4 Discussion

In the present study, we show that fibrosis, and in some cases even cirrhosis, can become less severe in AIH if properly treated and response to the treatment is good.

Cirrhosis was previously considered as an irreversible condition, but today this is no longer believed to be an absolute truth^[11,19]^ as studies on various liver diseases have shown that regression of fibrosis is possible.^[9–11,20,21]^ Liver fibrosis is probably a much more heterogenic and dynamic condition than was thought earlier with specific characteristics and prognoses for different diseases.^[19]^

This is also the main finding of our study where an improvement or at least stabilization of fibrosis grade from the first to the last biopsy was found in approximately 4 out of 5 patients. This result was valid even if biopsies taken at diagnosis or within the first year thereafter were excluded to avoid interference from lagging biochemical remission after start of medication^[22]^ and difficulties in interpreting the stage of fibrosis in the presence of acute inflammation. The finding of an improved fibrosis stage after medical treatment in AIH has been described previously,^[11–15,23]^ even if the study of Al-Chalabi et al^[24]^ did not find a significant reduction of fibrosis stage in a large cohort of AIH patients with a good treatment response.

We observed an association between the adherence to a continuous treatment with glucocorticoids and decreased or increased stages of fibrosis. Attempts to withdraw steroids were less often performed in patients with the decreased fibrosis stage (32.3% vs 81.3%, *P* = .002). The same pattern was not found for treatment with thiopurines. This finding might suggest that exacerbations of inflammation following drug withdrawal possibly may harm the liver, even if the medication is reintroduced. The beneficial effect of corticosteroids on liver histology in AIH has been described before and was partly attributed to the downregulating effect of glucocorticoids on TGF-β activation.^[12]^ The anti-fibrotic effects of second-line therapies (for example calcineurin inhibitors or MMF) are less studied, and AZA as mono therapy has not proven to reduce fibrosis.^[25]^

We also found in patients that were followed with repeated biopsies for more than 1 year that the absence of inflammation at last biopsy, that is an activity index score ≤3, was associated with a lower fibrosis stage, which is in line with previous studies.^[12,14,23]^

There are some limitations in our study design that should be addressed and considered when interpreting the results. The time range in which the biopsies were taken is wide and the reasons behind performing the biopsies are often unknown. Further, the patients included in the study were retrieved from a large cohort of Swedish AIH patients previously described by Werner et al.^[4,6]^ About 30% of the patients in the original cohort were found to have cirrhosis at diagnosis, and among the patients included in this study, we found 16 patients (16%) with cirrhosis at diagnosis. A plausible explanation for the discrepancy is that we only included patients that had at least 2 liver biopsies. Thereby, we naturally excluded patients with an aggressive disease, leading to transplantation or death close in time of the AIH diagnosis.^[14]^ Also, patients where the clinical value of performing a follow-up biopsy was questionable (for example older patients or patients that were considered to be too sick to have a second biopsy) would also naturally be excluded. Strengths of the studies are the large and well-defined cohort with long follow-up time, and that the number of paired biopsies is large.

Protocol biopsies are not performed in the clinical setting, but are most often performed at diagnosis and considered a prerequisite for diagnosis.^[1]^ In our study, we were able to retrieve biopsies from the time of diagnosis from 79 out 101 patients. In the clinical setting of AIH, biopsies are commonly performed within a couple of years after diagnosis, when transaminases are in sustained normalization, to assess fibrosis stage and remaining inflammation, and to thereby get an indication of prognosis. This strategy is also advocated in both international and national guidelines, as well as performing a biopsy when considering to stop treatment, when treatment seems insufficient and/or an overlap is considered.^[1,2,26]^ In the study, the bulk of biopsies was from the first years after diagnosis.

The risk of sampling error when assessing liver biopsies is well known,^[19,27,28]^ and the importance of a liver biopsy of an adequate size is known.^[28,29]^ A recently published review by Quaglia et al^[19]^ suggested that the risk for sampling error in untreated AIH is low, but is inevitable in advanced stages of liver disease due to a widespread heterogeneous pattern of damaged parenchyma mixed with preserved tissue. One should also keep in mind that the numerical system of stages of fibrosis might be misleading since a reduction in fibrosis from stage 4 to stage 2 does not equal a 50% decrease in fibrotic tissue.^[30]^

In conclusion, in this cohort of AIH patients primarily selected from primary catchment areas of university hospitals with a high frequency of medical treatment and a good treatment response, the histological response to medical treatment is good and fibrosis and cirrhosis are often reversed. The data suggest that a continuous low dose of corticosteroids may be beneficial. However, the point of no return of fibrosis is still not known in AIH and prospective studies with repeated liver biopsies are needed to understand when in the course of disease this occurs and to learn more about what characterizes patients who will not respond to therapy.
